# Assessment of Unexpected (Non-ABO) Red Blood Cell Antibodies and Their Associated Clinical Conditions Among Patients and Blood Donors Attending University Teaching Hospital of Kigali (CHUK) and Rwanda Blood Transfusion Division

**DOI:** 10.1155/ah/8871102

**Published:** 2025-03-19

**Authors:** Jean Baptiste Niyibizi, Daniel Seifu, Chelsey Geurkink, Erica Formiller, Thomas Muyombo, Christopher Gashaija, Henri Desire Uwayo, Gilbert Uwizeyimana, Laurie Gillard

**Affiliations:** ^1^School of Medicine, Basic Medical Sciences Division, University of Global Health Equity, Kigali, Rwanda; ^2^College of Health Sciences, Specialist in Blood Bank (SBB) Program, Rush University, Chicago, Illinois, USA; ^3^Versiti Illinois Immunohematology Reference Laboratory, Aurora, Illinois, USA; ^4^Blood Transfusion Division (BTD), Rwanda Biomedical Center (RBC), Kigali, Rwanda; ^5^Department of Pathology, University Teaching Hospital of Kigali (CHUK), Kigali, Rwanda

**Keywords:** immunohematology, transfusion, unexpected antibodies

## Abstract

Unexpected antibodies can cause hemolytic conditions. Therefore, screening for unexpected antibodies is essential for safe transfusion. The study was conducted at Rwanda Blood Transfusion Division and University Teaching Hospital of Kigali to assess unexpected antibodies with their associated clinical conditions. 8693 blood donors and 834 patients were screened for unexpected antibodies. Among 834 patients, 23 patients (2.75%) developed alloantibodies among which two of them had mixed alloantibodies. Five patients developed antibodies of uncertain specificities. Among 8693 blood donors, only 4 blood donors (0.046%) had clinically significant alloantibodies, whereas 6 blood donors (0.069%) had antibodies of uncertain specificities. Moreover, 3 patients (0.35%) had autoantibodies in their plasma. Different types of anemia were presented with patients who developed unexpected alloantibodies. History of transfusion and pregnancy were predictors of alloimmunization among patients (*p* < 0.01). Antibody screening and antibody identification are important for safe blood transfusion practices.

## 1. Introduction

Different populations vary in the prevalence of significant blood antibodies, and thus safe blood transfusion requires accurate blood screening and compatibility testing [1]. Blood compatibility testing is necessary to prevent critical complications during blood transfusion. The Association for Advancement of Blood and Biotherapies (AABB) has recommended that ABO typing, Rh typing, unexpected antibody screening, and cross-matching must be performed before a blood transfusion. However, in case of an emergency in which the unexpected antibody screening test is not done, the delay in finding compatible blood may be life threatening [2]. The most potent immunogenic antibodies responsible for red cell lysis are in the order: ABO system > Rh system > Kell system > Kidd system. Alloantibodies are circulating antibodies produced as a result of earlier antigenic stimulation from blood transfusions or pregnancy. Autoantibodies are endogenous antibodies that an organism makes in response to a component of its own tissue [2].

Unexpected antibodies, defined as any non-ABO antibodies present in recipient or donor, cannot be confirmed to exist in a person's serum until tested. Most of the unexpected antibodies are not “naturally occurring” as those of the ABO system. Note that individuals produce antibodies against the ABO system antigens without stimulation by foreign red cells. Unexpected antibodies are, instead, produced in response to stimulation by exposure to foreign antigens during red cell transfusion or during pregnancy (alloimmunization). Once these immune antibodies are formed, they can lead to hemolytic disease of the fetus and newborn (HDFN) as well as hemolytictransfusion reactions (HTR). Because immune antibodies persist for a lifetime, it is essential to account for them in blood transfusions and closely monitor them during pregnancy [3]. Unexpected antibodies to antigens of highest clinical relevance are from systems including Rh, MNSs, Duffy, Kidd, Kell, Lewis, Lutheran, and Xg [3]. Alloimmunization is an immune reaction to foreign antigens from another person, which most typically occurs following pregnancy or blood transfusions. In certain circumstances, the body comes into contact with foreign cells that contain specific antigens, or proteins on the cell surface that might elicit an immunological response. When these antigens are recognized, white blood cells develop antibodies, which signal the immune system to eliminate the antigen-containing foreign cell. Typically, this process protects the body from harmful foreign organisms such as germs. When this occurs in response to another person's blood products, it is known as alloimmunization, and it can lead to serious complications [4].

Unexpected antibodies can cause transfusion reactions including acute or delayed HTR or neonate hemolytic syndrome [4]. A factor impacting unexpected alloimmunization rate has been linked to transfusion and pregnancy and has been documented to be 1%–10% and even higher (20%) in regularly transfused populations such as individuals with sickle cell disease or thalassemia [4]. Unexpected antibodies are typically of the IgG and cannot be detected in normal ABO typing or crossmatch. Pretransfusion testing, particularly antibody screening, has been developed to identify these antibodies, and antibody detection techniques are designed to detect different antibodies [5–7]. More properly called the “antibody detection test,” the antibody screen (as most blood bank personnel call it and as most blood bank types call it) is a test used to demonstrate the presence or absence of “unexpected (non-ABO) antibodies.” It is used as a preliminary test to predict whether the patient has antibodies that could be incompatible with donor red blood cells (RBCs) [6, 7].

A study conducted in Rwanda examined the risk of RBC alloimmunization associated with immediate spin crossmatch versus the antiglobulin crossmatch in patients receiving blood transfusions. The findings indicated that the rate of red cell alloimmunization was higher in hospitals using immediate crossmatch compared to those using the antiglobulin crossmatch. As a result, the study recommends favoring antiglobulin crossmatch over immediate spin crossmatch [8]. In many African countries, including Rwanda, there is no testing for unexpected antibodies prior blood transfusion for a patient. This could be causing the transfusion of incompatible blood to patients due to unexpected antibodies that are usually nondetectable during ABO and Rh blood crossmatch, and this could lead to delayed HTRs. No single study has been conducted in Rwanda on antibody screening, detection, and identification. This research aims to determine the occurrence of unexpected antibodies in blood donors and patients attending the Blood Transfusion Division (BTD) and the University Teaching Hospital of Kigali, along with their associated clinical conditions.

## 2. Research Methodology

### 2.1. Study Design and Participants

This cross-sectional study assessed the frequency of unexpected antibodies among blood donors and patients attending BTD and University Teaching Hospital of Kigali (Centre Hospitalier Universitaire de Kigali _ CHUK). The included blood donors were individuals aged 18 and above, weighing 50 kg and above, and who have been screened negative for key major infectious diseases per BTD guidelines. Patients included in this study were those whom blood transfusion products were requested by clinicians during the study time period (from 24^th^ August 2022 to 30^th^ November 2022). The sample size was calculated using the formula:(*n* = *Z*_*α*/2_^2^∗*p*∗(1 − *p*)/*e*^2^)[9], where *Z*_*α*/2_ is the critical value in the normal distribution at *α*/2 (for example, with a 95% confidence level, *α* is 0.05 and the critical value is 1.96). The margin of error is referred to as *e* (0.05 or 5%). p is the estimated proportion for the outcome (since proportion is unknown, 50% is used). For this current research, *e* was set at 3.39% for patients (*n* = 834) and 1.05% for blood donors (*n* = 8693).

During the study period, we used a nonprobability convenient sampling technique to select all participants who met the eligibility criteria. All necessary information was captured in the data collection tool, and these included gender, history of blood transfusion, history of pregnancy, and the current (present or observed) clinical diagnosis. Data were collected from blood donor form and patient blood request form.

### 2.2. Antibody Screen and Identification

Monoclonal antibodies, ABO cells (A1 cells and B cells), panoscreen cells (I, II, and III), 10 panel cell identification, and 16 panel cell identification were sourced from Immucor, Inc. (acquired by Werfen in 2022), Norcross, GA, USA [10].

Plasma samples were tested against three individual screening cells: R1R1, R2R2, and rr. These screening cells were Group O and included common antigens relevant to the manufactured population, such as D, C, c, E, e, K, k, Kpa, Kpb, Jsa, Jsb, Fya, Fyb, Jka, Jkb, Lea, Leb, P1, M, N, S, s, Lua, Lub, and Xga. All blood donor samples were screened for unexpected antibodies using the QWALYS automated analyzer (DIAGAST Ltd, France), which employs a solid-phase serology assay using Erythrocyte Magnetized Technology (EM) (DIAGAST, 2022) [10]. For the samples in which the cells were nonreactive based on either tube- or solid-phase technology, it is more accurate to describe the result as “antibody detection negative” (no antibodies detected) rather than “antibody screen negative” (indicating the absence of antibodies). This distinction is important because no test, including those used in this study, is completely foolproof; thus, significant antibodies—especially those targeting rare red cell antigens—may go undetected in standard tests [6].

All positive antibody screened samples were subjected to antibody identification with 11 panel cells. Check cells were employed to validate antihuman globulin (AHG) functionality during the indirect antihuman globulin (IAT) phase. Samples were ruled out on the common antibodies for the following antigens: D, C, c, E, e, K, k, Kpa, Kpb, Jsa, Jsb, Fy^a^, Fy^b^, Jk^a^, Jk^b^, Le^a^, Le^b^, P_1_, M, N S, s, Lu^a^, Lu^b^, and Xg^a^.

### 2.3. Statistical Analyses

Descriptive statistics were computed using STATA Version 18. Quantitative data analysis entailed descriptive/univariate, bivariate, and multivariable analyses. Descriptive analyses were computed using frequency and percentages to determine the prevalence of alloantibodies among study participants. In bivariate analysis, a chi-square test was applied to assess the association between dependent and independent variables. Multivariable analysis was performed to control confounding variables and determine the strength of the association between the development of alloantibodies and clinical conditions with the assumption that most clinical significant human alloantibodies are due to immunization through transfusion exposure. The significance level was set at 5%. The data are presented using tables. Descriptive statistics were used in the statistical analysis. The results of the analysis were presented in tables.

### 2.4. Ethical Considerations

This research was conducted after obtaining ethical approval from the Institutional Review Board (IRB) of the University of Global Health Equity (UGHE) (Protocol Ethical Number: 189) and subsequent authorization approvals from the Rwanda Biomedical Center and University Teaching Hospital of Kigali (Ethical Number: Ref: EC/CHUK/121/2022). Written consent was obtained from blood donor participants prior to taking part in this research. No minor blood donors participated in this research. Consent from patients was waived by the research ethics committee since the researchers used the samples that were collected for routine transfusion requests such as ABO, RH blood group typing, and crossmatch.

## 3. Results

The results describe the frequency of unexpected alloantibodies in both nonrenumerated new and repeat donors who participated in this study. The results also depict the frequency of alloantibodies in patients who needed blood transfusion. Furthermore, the results illustrate the association of alloantibody development versus clinical and demographic characteristics.

### 3.1. Unexpected Antibody Screening and Identification in Patients

As illustrated in Table 1, upon identification, 23 patients (2.75%) were tested positive for single alloantibodies, and among these, two patients had multiple antibodies. The study found the following frequencies of antibodies: 12 in the Rh system, four in the Duffy system, three in the Kell system, three in the MNS system, two in the Kidd system, and one in the Lewis system. In addition, there were no antibodies identified in the P, Lutheran, and Xg blood systems. Two patients showed mixed antibody reactivity suggesting anti-Fyb and anti-M, and another patient appeared to have developed anti-Fy^a^ and anti Jk^a^.

### 3.2. Prevalence of Unexpected Antibodies in Blood Donors

The total screened donors were 8693.

Results from Table 2 show that significant alloantibodies were found in 4 blood donors (0.046%). One blood donor had mixed antibodies (anti-D and anti-C). The 6 blood donors (0.069%) had antibodies of uncertain specificities.

**Figure 1:**
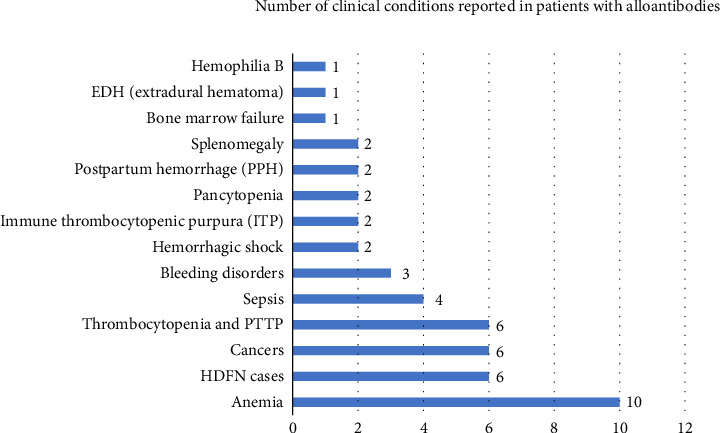
Clinical conditions reported in patients with alloantibodies. This figure describes the frequency of alloantibodies in patients in relation to their clinical presentations or diagnosis prior transfusion.

The results from Figure 1 show that the predominant clinical condition associated with alloantibody development was anemia, followed by HDFN cases, cancers, and thrombocytopenia.

The results from Table 3 show that most antibodies were found in patients with blood group O Rh negative.

Table 4 demonstrates that alloantibodies were associated with history of transfusion, pregnancy, and gender.

Table 5 demonstrates that the development of antibodies in nonpregnant women is likely due to previous exposure to blood transfusions (AOR: 13.94, CI: 1.98–189.54, *p* = 0.048).

## 4. Discussion

According to the AABB Standards, 2020, Technical manual 5.14 [6], pretransfusion tests for allogeneic transfusion shall include ABO group and Rh type. In addition, for whole blood, RBC, and granulocyte components, pretransfusion testing for unexpected antibodies to RBC antigens shall be performed. After the antigens of the ABO and Rh blood group systems, the K antigen is the antigen that causes the greatest immune response [7, 11]. Adverse transfusion reactions and HDFN can be brought on by antibodies that are directed against the Kell antigens. ABO and Rh incompatibilities are the most frequent causes of HDFN. However, ABO HDFN is typically mild to moderate, and HDFN due to anti-Rh disease is largely preventable through the use of RhIG prophylaxis [7].

In Rwanda, although antibody screening is done for donors, there is no antibody identification that is done for these donors who are screened positive for the presence of unexpected red cell antibodies. To prevent alloimmunization in Rwanda, the Rwanda BTD has started testing for the K antigen in blood donors and the K+ blood units are discarded, and the blood donors are permanently deferred. However, according to standards, currently some consider RBCs with known unexpected antibody acceptable for transfusion. The transfusable unit must be labeled with identified antibodies as required by AABB standards [6] so that appropriate compatible unit will be selected prior to crossmatch and transfusing a patient. For instance, units of blood that are screened positive for the Kell antigen should be transfused to a patient who does not have anti-K antibodies or who has Kell antigens. This would increase donor repository of available red cells [7].

In the current study, the most common identified alloantibodies among screened patients were anti-D (0.71%) of the Rh system. In whole, the Rh system represented a high frequency of the antibodies identified (1.43%) in the current study. The Duffy system followed with a frequency of 0.47%, while both the Kell and MNS systems had a frequency of 0.35%. The Kidd system was found in 0.23% of cases, and the Lewis system had the lowest frequency at 0.11% of alloimmunization. Only two patients had a mixed alloantibody (Table 1). In blood donors, Rh system represented a prevalence of 0.057% with anti-D at 0.034% (Table 2). In this study, one blood donor had multiple antibodies that are anti-C and anti-D. Although it was not confirmed, this could indicate the presence of the anti-G in this female blood donor. Antibody of uncertain specificity (AUS) was high in blood donors (0.069%).

The findings of this current study agreed with many studies conducted around the globe where the most antibodies reported were in Rh-Hr blood group system. In the study done by Gupta et al. [12] on antibody screening and identification in Western India, the irregular alloantibodies were found in 512 patients and 11 donors and the most common alloantibody found was anti-D (0.075%) and the clinical conditions most associated with alloantibody development in these patients were antenatal cases followed by thalassemia, severe anemia, sickle cell anemia, congenital heart diseases, preoperative anemia correction, trauma, and HDFN.

In the study done by Waggiallah et al. [13] on the prevalence of unexpected antibodies in Saudi Arabian patients, the frequency of unexpected antibodies was 107 (1.1%), with the predominant being Rh system. These antibodies were associated with clinical conditions such as history of transfusion, pregnancy, and autoimmune diseases. The findings of this study agreed with the present study where some patients who were identified to have alloantibodies had history of transfusion and pregnancy (Table 4) with strong statistical association (*p* = 0.005) (Table 6). Additionally, the results of the current study indicated that nonpregnant women who developed alloantibodies likely did so due to previous transfusion episodes. However, there is also a possibility of alloimmunization occurring from very early pregnancies (AOR: 13.94, CI: 1.98–189.54, *p* = 0.048) (Table 5).

In the study done by Chen et al. on the screening of unexpected antibodies in donor and patients receiving transfusion in China, it was found that the overall prevalence of unexpected antibodies was approximately 0.2% [1] and 1548 participants presented with HDFN, mostly due to anti-D. The findings of this study agreed with the current study, where the cases of HDFN were mostly due to anti-D. In the study done by Makroo et al. [14] on antibody screening and identification in patients at a tertiary care hospital in New Delhi, the overall alloimmunization rate was 0.49% and the most common identified alloantibody was anti-E (37.2%), followed by anti-D (19.2%). Contrary to this, the rate of alloimmunization against antigen E in the current study was 0.59% in patients while it was 0% in donors.

In the study done by Ko et al. [15] on frequency of unexpected antibody and consideration during transfusion, the frequency of unexpected antibodies was 1.52% and 51.4% of the participants had a history of pregnancy and delivery, while 20.2% had a history of transfusion. This study found a lot of mixed alloantibodies cases that were different from the current study. In the study done by [16], the frequency of unexpected antibodies was 3.4% among pregnant women. The specificity of the antibodies was as follows: anti-C 6 (1.2%), anti-E 3 (0.6%), anti-Jsb 3 (0.6%), and anti-K 5 (1.0%). There was no anti-D identified despite 8.6% of the study population being Rhesus D (Rh D) negative. This could suggest that management of anti-D cases' alloimmunization is well controlled in Rh-negative pregnant mothers. In the study done by [17] on the frequency of unexpected antibodies in Nanjing, the overall frequency of alloimmunization was 32.5% with Rh system being the most frequent. This study revealed extreme high alloimmunization (32.5%) compared to the current study.

In the current study, the majority of antibodies were found in patients who were suffering from different types of severe anemia, followed by thrombocytopenia cases, HDFN, and cancer among others (Figure 1). In the current study, anti-D was the most powerful antibody associated with HDFN. ABO HDFN is usually mild because the ABO antigens are weakly developed in fetus and subsequently the antigen structures slowly grow and mature over the first few years of life. Although most anti-ABO are IgM that cannot traverse placenta, some are IgG and can traverse placenta and cause HDFN. Many anti-A and anti-B that are IgG are found in group O patients. IgG class I is the most potent in causing HDFN, whereas IgG class II is the least in causing HDFN. In nature, IgM are highly molecular and cannot cross the placenta. It is also worth noting that the major immunization occurs at delivery where there is potential mixture of fetal–mother blood. While antibodies to Rh antigens are not “naturally occurring,” the antigens of the Rh system are highly immunogenic (capable of causing an immune response). The immunogenicity is in the order D> c > E > C > e.

Another antibody identified in this study was anti-K which causes severe HDFN. Kell antigens are present on myeloid progenitor cells, and anti-K suppresses fetal hematopoiesis. Anti-M and Anti-N rarely result in HTRs or HDFN. Most of anti-M and anti-N are IgM and few are IgG. Anti-S and anti-s can cause acute or delayed transfusion reactions and HDFN. Anti-Fy^a^ and anti-Fy^b^ can cause mild acute or delayed HTRs and HDFN. In the current study, anti-Le^a^ was found with strong reactivity. Anti-Le^a^ is very common in black and pregnant women. Most of anti-Le^a^ are naturally occurring and many of them are IgM and IgG. Anti-Le^a^ rarely cause HTRs, and they never cause HDFN because there is no Lewis antigen expressed on cord cells. Anti-C^w^ is an IgG with rare cases of IgM and usually occurs naturally, although it may also be immune stimulated, either through transfusion or pregnancy-related RBC exposure. It often occurs in association with other alloantibodies.

It was found in the current study, among patients, that many alloantibodies were in blood group O, where O+ represents 40.90% and O− represents 31.81% of the positive cases (Table 3). Anti-D were found in RhD-negative mothers, and this is an obvious alloimmunization due to inheritance of RhD antigen from the father to the fetus. In addition, for pregnant mothers, they might be group O+ but lack a wide range number of other antigens found in different group systems and thus when married to husband with whom those antigens are expressed, half of it may be passed on to the offspring and eventually lead to alloimmunization during pregnancy.

Among blood donors, it was found that more clinically significant alloantibodies were found in the blood group O− (20%) and O+ (30%) of the positive cases (Table 3). In this study, all positive cases were found in patients who were previously transfused or women with history of pregnancy or delivery. It was also found that among patients, the alloantibody frequency was higher in females (77.27%) than males (22.73%) (Table 4) and could be due to pregnancy.

Autoantibodies were identified in 3 participants, two of whom had blood cancer and one with hyperactive splenomegaly. It is believed that detectable autoantibodies in plasma could be a predictable marker for cancer development in a patient [18]. Genetics, epigenetics, and environmental factors are believed to contribute to autoimmune disorders [19]. In the current study, it was difficult to differentiate autoantibodies in order to determine whether they are combined with other alloantibodies or if they are singly occurring, due to lack of autoadsorption reagents.

Single nucleotide polymorphism (SNPs) are responsible for variations in RBC antigens. These SNPs can be detected using various DNA sequencing methods, including high-resolution sequencing, targeted sequencing, less targeted sequencing, and untargeted sequencing [20–22]. In some countries including Rwanda, serology is still considered the backbone for this weak D antigen identification. In the future, the introduction of high-resolution molecular genotyping will improve the certainty of confirming weak D antigen as it is even a recommendation from AABB standards for blood donor centers [6]. Molecular genotyping is considered the gold standard for typing clinically significant antigens, including those used in tissue matching, chronic transfusion anemia, and platelet antigens for which serological methods have not yet been established. Extended antigen profiling or typing is required to expedite future pretransfusion testing by narrowing the number of different antibody specificities that must be ruled out to include only those antigens that the patient lacks [23, 24]. In addition, extended typing is and has been proven to be of great assistance in managing different conditions such as sickle cell disease patient, patient receiving monoclonal antibodies therapy, and patients with autoimmune hemolytic anemia [25]. Nucleic acid–based testing offers several advantages over protein-based methods for typing patients who have recently received transfusions. It is particularly useful for determining paternal zygosity for RHD and Human Platelet Antigens (especially HPA-1), as well as for antenatal fetal typing. This testing can aid in the early detection of HDFN and Fetal and Neonatal Autoimmune Thrombocytopenia (FNAIT) [25].

As most cases of anti-D in this study were found in mothers with history of pregnancy, the management of RhD-negative women is critical to prevent alloimmunization, especially for the prevention of HDFN. The most feasible approach being implemented to prevent HDFN in most countries, including Rwanda, involves the administration of RhIg. Based on the findings of the current study, there is a need to continue improving timely follow-up of RhD-negative pregnant mothers and to accurately administer the right RhIg dose. Again, improving awareness about human blood groups, blood donation, blood transfusion, premarital blood screening,and prenatal screening through public education could support this practice. Moreover, though it is not yet widely implemented, using cell-free fetal DNA (cffDNA) allows for the easy determination of RhD blood type of the fetus without the need for invasive procedures such as cordocentesis. This noninvasive approach enables healthcare providers to recommend the administration of Rh Immune Globulin (RhIG) when necessary [26–29] [30–32]. In 2015, a group work representing a number of clinical standards organizations recommended that RhD genotyping be incorporated in the management of pregnant women [33, 34].

For blood donor centers, sometimes weak D might be labeled as RhD negative, whereas it is RhD + while using serology. Though some centers (including US) label weak D as D positive, it is better recommended that weak D be confirmed using genotyping methods [21]. High resolution and less targeted nucleic acid testing such as next generation sequencing is a powerful tool for transfusion medicine, allowing for the identification of rare and novel blood group variants that would otherwise be unrecognized particularly when the antibody specificity is uncertain or, importantly, when transfused RBC survival is compromised in the absence of detectable antibody [35, 36].

In some countries or some blood transfusion centers and institutions with regular shortage of Rh antigen negative blood products, in the context of emergent transfusion, all males and females who are not of childbearing age receive ORhD + blood products, while females of childbearing potential who have an unknown blood type receive type O RhD negative transfusions to prevent sensitization to the D antigen. While this could serve as an alternative option, it may not be the most effective choice. There is a risk of alloimmunization due to other Rh antigens or from different blood group systems, which could lead to HDFN in females of childbearing age [37–40].

## 5. Conclusion

The prevalence of significant alloimmunization among the studied population is 2.75% in patients and 0.046% in blood donors. The development of unexpected alloantibodies was strongly associated with previous transfusion exposures, history of pregnancy, or deliveries in Rh-negative mothers (*p* < 0.001). It is therefore recommended that antibody screening and antibody identification are carried out especially among patients with history of pregnancy and multiple transfusions. Pregnant women who are RhD negative should be well monitored throughout pregnancy till delivery and post-delivery by providing adequate RhIG appropriately.

## Figures and Tables

**Table 1:** Prevalence of unexpected antibodies in 834 patients.

**Identified antibodies**																							
	**RH-Hr**						**Kell**		**Duffy**		**Kidd**		**Lewis**		**P**	**MNS**				**Lutheran**		**Xg**	**Total**
	**> D**	**> C**	**> c**	**> E**	**> e**	**> ** **C** ^**w**^	**> K**	**> k**	**> ** **F** **y** ^**a**^	**> ** **F** **y** ^**b**^	**> ** **J** **k** ^**a**^	**> ** **J** **k** ^**b**^	**> ** **L** **e** ^**a**^	**> ** **L** **e** ^**b**^	**> ** **P**_1_	**> M**	**> N**	**> S**	**> s**	**> ** **L** **u** ^**a**^	**> ** **L** **u** ^**b**^	**X** **g** ^**a**^	

	6	1	0	5	0	0	3	0	3	1	1	1	1	0	0	1	0	2	0	0	0	0	25
%	0.71	0.11		0.59			0.35		0.35	0.11	0.11	0.11	0.11			0.11		0.23					2.9

	12 (1.43%)						3 (0.35%)		4 (0.47%)		2 (0.23%)		1 (0.11%)		0	3 (0.35%)				0		0	25 (2.9%)

*Note:* The table shows the frequency of irregular antibodies identified among 834 patients. Frequency of autoantibodies: 3 (0.35%). Antibodies of uncertain specificities: 5 (0.59); antibodies against high and low antigens, unable to be specified.

**Table 2:** Prevalence of irregular antibodies in 8693 red cell donors.

**Identified antibodies in 8693 donors**																							
	**RH-Hr**						**Kell**		**Duffy**		**Kidd**		**Lewis**		**P**	**MNS**				**Lutheran**		**Xg**	**Total**
	**> D**	**> C**	**> c**	**> E**	**> e**	**> ** **C** ^**w**^	**> K**	**> k**	**> ** **F** **y** ^**a**^	**> ** **F** **y** ^**b**^	**> ** **J** **k** ^**a**^	**> ** **J** **k** ^**b**^	**> ** **L** **e** ^**a**^	**> ** **L** **e** ^**b**^	**> ** **P**_1_	**> M**	**> N**	**> S**	**> s**	**> ** **L** **u** ^**a**^	**> ** **L** **u** ^**b**^	**> ** **X** **g** ^**a**^	

Number	3	1	0	0		1	0	0	0	0	0	0	0	0	0	0	0	0	0	0	0	0	5
Percentage	0.034	0.011				0.011																	

	5 (0.057%)						0		0		0		0			0				0			

*Note:* The table shows the frequency of irregular antibodies among blood donors. Frequency of antibody of uncertain specificity: 6 (0.069%).

**Table 3:** Distribution of antibody screening results within the blood groups among patients and blood donors.

Patients			Blood donors		
ABO/RhD	Number of cases	Identified alloantibodies	ABO/RhD	Number of cases	Identified antibodies
A+	3 (13.63%)	anti-Fy^b^ and anti M, anti-K	A+	2 (20%)	Antibody of uncertain specificity (AUS)
A−	0		A−	1 (10%)	Anti-D,
B+	3 (13.63%)	Anti-E, anti-S,	B+	2 (20%)	AUS
B−	0		B−	0	None
AB+	1 (4.54)	Anti-C	AB+	0	None
AB−	0		AB−	0	None
O+	9 (40.90)	Autoantibodies, anti-K, anti-E, anti-S, anti-K, anti-Fy^a^, anti-Jka, and anti-Jkb	O+	3 (30%)	Anti-C^w^, AUS
O−	7 (31.81)	Anti-D, anti-Le^a^	O−	2 (20%)	Anti-D and anti-C

*Note:* The table shows alloantibodies and corresponding blood groups in which they were found.

**Table 4:** Cases according to sex, transfusion history, and pregnancy among patients.

	Condition		Number of cases	Identified antibodies
1	History of previous transfusion		16	Autoantibodies, anti-E, anti-K, anti-S, anti-Fy^b^, anti-C, anti-Le^a^, anti-Jk^a^

2	History of pregnancy and delivery		7	Anti-D and anti-E

3	Gender	Males	5 (21.73%)	Anti-S, anti-E, anti-C, anti-JKb
		Females	18 (78.26%)	Anti-K, anti-D, anti-S, anti-E, anti-Fy^b^, anti-Fy^a^, anti-Manti-Le^a^, anti-Jka

*Note:* The table demonstrates the number of antibodies according to presented clinical cases.

**Table 5:** Multivariable analysis of outcome variables associated with development of unexpected alloantibodies.

History of pregnancy and delivery	AOR⁣^∗^	95% CI		*p* value
		Lower	Upper	
Yes	1			
No	13.94	1.98	189.54	0.048

^∗^AOR, adjusted odds ratio (predicted probabilities are of membership of being ever transfused).

**Table 6:** Statistical association of risks and clinical conditions associated with development of unexpected alloantibodies other than transfusion exposure.

Variables	*N*	Ever transfused		Chi-2 *p* value
		No	Yes	
*Sex*				
Male	5	1 (20.0)	4 (80.0)	0.567
Female	18	6 (33.3)	12 (66.7)	

*History of pregnancy and delivery*				
Yes	7	5 (71.43%)	2 (28.57)	**0.005**
No	16	2 (12.5%)	14 (87.5%)	

*Anemia*				
Yes	16	11 (68.75)	5 (31.25)	0.074
No	7	2 (28.57)	5 (71.43)	

*Antenatal cases (HDFN, jaundice)*				
Yes	17	2 (33.33)	4 (66.67)	0.858
No	6	5 (29.41)	12 (70.59)	

*Cancers*				
Yes	6	1 (16.67)	5 (83.33)	0.394
No	17	6 (35.29)	11 (64.71)	

*Thrombocytopenia and PTTP*				
Yes	6	2 (33.33)	4 (66.67)	0.858
No	17	5 (29.41)	12 (70.59)	

*Sepsis*				
Yes	4	1 (25)	3 (75)	0.795
No	19	6 (31.58)	13 (68.42)	

*Bleeding disorders*				
Yes	3	2 (66.67)	1 (33.33)	0.144
No	20	5 (25)	15 (75)	

*Hemorrhagic shock*				
Yes	2	0	2 (100)	0.328
No	21	7 (33.33)	14 (66.67)	

*Immune thrombocytopenia purpura*				
Yes	2	1 (50)	1 (50)	0.529
No	21	6 (28.57)	15 (71.43)	

*Pancytopenia*				
Yes	2	0	2 (100)	0.328
No	21	7 (33.33)	14 (66.67)	

*Postpartum hemorrhage (PPH)*				
Yes	2	0	2 (100)	0.328
No	21	7 (33.33)	14 (66.67)	

*Splenomegaly*				
Yes	2	0	2 (100)	0.328
No	21	7 (33.33)	14 (66.67)	

*Bone marrow failure*				
Yes	1	0	1 (100)	0.499
No	22	7 (31.82)	15 (68.18)	

*Hematoma*				
Yes	1	1 (100)	0	0.122
No	22	6 (27.27)	16 (72.73)	

*Hemophilia*				
Yes	1	0	1 (100)	0.499
No	22	7 (31.82)	15 (68.18)	

*Note:* Bold value indicates statistically significant (*p* < 0.05).

## Data Availability

All raw data and other processed data can be obtained from the corresponding author upon request.
